# Initial report of the osteogenesis imperfecta adult natural history initiative

**DOI:** 10.1186/s13023-015-0362-2

**Published:** 2015-11-14

**Authors:** Laura L. Tosi, Matthew E. Oetgen, Marianne K. Floor, Mary Beth Huber, Ann M. Kennelly, Robert J. McCarter, Melanie F. Rak, Barbara J. Simmonds, Melissa D. Simpson, Carole A. Tucker, Fergus E. McKiernan

**Affiliations:** Division of Orthopedics and Sports Medicine, Children’s National Health System, 111 Michigan Ave NW, Washington, DC 20010 USA; Osteogenesis Imperfecta Foundation, Gaithersburg, MD USA; Rehabilitation Institute of Chicago, Chicago, IL USA; Morris Animal Foundation, Denver, CO USA; Temple University, Philadelphia, PA USA; Marshfield Clinic, Chippewa Falls, WI USA

**Keywords:** Osteogenesis imperfecta, PROMIS®, Patient centered outcomes, Childhood onset conditions, Adult

## Abstract

**Background:**

A better understanding of the natural history of osteogenesis imperfecta (OI) in adulthood should improve health care for patients with this rare condition.

**Methods:**

The Osteogenesis Imperfecta Foundation established the Adult Natural History Initiative (ANHI) in 2010 to give voice to the health concerns of the adult OI community and to begin to address existing knowledge gaps for this condition. Using a web-based platform, 959 adults with self-reported OI, representing a wide range of self-reported disease severity, reported symptoms and health conditions, estimated the impact of these concerns on present and future health-related quality of life (QoL) and completed a Patient-Reported Outcomes Measurement Information System (PROMIS®) survey of health issues.

**Results:**

Adults with OI report lower general physical health status (*p* < .0001), exhibit a higher prevalence of auditory (58 % of sample versus 2–16 % of normalized population) and musculoskeletal (64 % of sample versus 1–3 % of normalized population) concerns than the general population, but report generally similar mental health status. Musculoskeletal, auditory, pulmonary, endocrine, and gastrointestinal issues are particular future health-related QoL concerns for these adults. Numerous other statistically significant differences exist among adults with OI as well as between adults with OI and the referent PROMIS® population, but the clinical significance of these differences is uncertain.

**Conclusions:**

Adults with OI report lower general health status but are otherwise more similar to the general population than might have been expected. While reassuring, further analysis of the extensive OI-ANHI databank should help identify areas of unique clinical concern and for future research. The OI-ANHI survey experience supports an internet-based strategy for successful patient-centered outcomes research in rare disease populations.

**Electronic supplementary material:**

The online version of this article (doi:10.1186/s13023-015-0362-2) contains supplementary material, which is available to authorized users.

“Progress can be made when patients and researchers collaborate towards a common goal [1].”

## Background

Osteogenesis imperfecta (OI) is a heterogeneous group of inherited connective tissue diseases defined clinically by excessive skeletal fragility and recurrent fracture [2]. With an estimated prevalence of 8 cases per 100,000 persons, perhaps 30,000 live with OI in the United States [2]. The Sillence classification for OI, first described in 1979, includes four types (I-mild, II-neonatal lethal, III-severe, progressively deforming, and IV moderately severe), and is almost exclusively based on physician perception of disease severity [3].

Most OI cases are caused by mutations in the type I collagen genes and are the result of autosomal dominant inheritance. During the past several years, the spectrum of OI has expanded to include rare, recessively inherited forms resulting from abnormalities in the post-translational modification of type I collagen, as well as abnormalities in regulatory proteins involved in bone matrix homeostasis, including those of the Wny-signaling pathway [4, 5]. These non-classical forms of OI can range from moderate severity to neonatal lethality in presentation.

While the cardinal clinical manifestation of OI is fracture [2], deficiencies in collagen-rich tissues other than bone can lead to abnormal dentition [6], joint laxity [7, 8], hearing loss [9], ocular disease [10], pulmonary [2], and vascular and valvular heart disease [11]. Disease severity ranges from perinatal death to minimally symptomatic forms that escape detection into adulthood [2]. Heightened diagnostic awareness and improved treatments, particularly in severe forms, has increased the number of adults living with OI [12]. While adolescents and young adults with OI may be medically sophisticated, their transition to a new adult health care provider and system of care can be daunting. Adult health care providers and systems have a paucity of medical evidence to guide them and likely little first-hand experience with this condition [13]. Similarly, physically accessible, high quality, informed health care may not be readily available to many [14] and some adults with OI feel that the strong association of OI with “brittle bones” diverts attention away from other important adult health care concerns.

Limited research exists regarding the health issues of adults with OI [7, 8]. Although adults with OI report a high level of life satisfaction, any conclusions drawn from available reports are constrained by the small number of subjects, the focus on milder forms of OI, and the absence of disease specific quality of life (QoL) measures. Thus, adults with OI, their families and their health care providers cannot formulate expectations of their future health-related QoL, nor anticipate their long-term health care needs. Moreover, despite the potential for mortality and significant morbidity suffered by OI patients, all measures of OI outcomes have been developed by experts without input from patients and rely on clinician-based assessments rather than actual patient reports.

Directly engaging the OI community in patient-centered outcomes (PCO) research could mitigate some of these challenges [1, 15]. To address concerns raised by the adult OI community and to begin closing the knowledge gap, the Osteogenesis Imperfecta Foundation (OIF) assembled a committee composed of adults with OI (including a practicing physician), orthopedic and medical specialists with extensive experience in the care of persons with OI, OIF representatives, research scientists, and statisticians and created the Osteogenesis Imperfecta Adult Natural History Initiative (OI-ANHI) in 2010. The OI-ANHI tested the feasibility of using web-based, PCO methods for rare disease research by creating an on-line health survey to (1) define health care concerns and perceptions of adults with OI; (2) identify health-care related issues that may have been previously missed or under-valued by adults with OI and their medical providers; and (3) compare QoL responses by adults with OI with those of benchmark populations without OI. This paper reports the initial results of the OI-ANHI survey.

## Methods

Based on adult focus group meetings conducted by OI-ANHI committee members at the 2010 bi-annual OIF meeting for persons with OI and their families, a web-based survey was designed and implemented to capture the health status, concerns, needs, and priorities of adults with OI. The complete OI Adult Natural History Initiative survey is available as Additional file 1. The survey was presented in the following sequence: (1) collection of general anthropomorphic, demographic, and medical information; (2) an organ-system-based, medical “review of systems” (ROS) followed by a 5-point Likert ranking to assess the impact of each organ-system on current and expected future QoL; (3) multiple instruments from the Patient-Reported Outcomes Measurement Information System (PROMIS®) to evaluate the level of health and functioning for core physical, mental, and social health constructs that are normed to the US general population [16].

Anthropometric data included height and weight. Demographics included year of birth, gender, race/ethnicity, education level, and current country of residence. Medical information addressed medical diagnoses, testing and interventions, and various lifestyle factors. Data collected include OI Sillence Classification, additional medical diagnoses, primary means of mobility, list of health care providers, surgical health interventions, and level of physical activity among other variables.

We queried severity of OI in three different ways: first, respondents provided their understanding of their Sillence type; secondly, they reported whether they perceived their disease to be mild, moderate or severe; height provided an objective measure that has been shown to correlate with disease severity [17].

The second portion of the survey was a medical review of systems (ROS), developed as a collaborative effort by the group of authors, which included physicians, persons with OI, and their advocates, yielding a comprehensive, textbook-quality medical ROS and potentially identifying previously underemphasized or unidentified health concerns. (Additional file 1) We assessed respondents’ physical health within fifteen organ systems: neurologic, urinary, musculoskeletal, auditory, cardiovascular, skin, vision, dental, pulmonary, gastrointestinal, endocrine, hematologic, oral, obstetric/gynecologic (females only), and male sexual function (males only). We then used a tri-part format to assess the impact of each system on the respondent’s QoL. The first question asked whether or not the respondent had any of the concerns listed specific to the system (e.g. for the skin, rashes and pressure sores; for the pulmonary system, shortness of breath, pneumonia, etc.). The second and third questions for each organ system queried respondents via a 5-point, Likert rating scale as to how much issues within the relevant organ system currently impacted their QoL and how much they thought problems with each system would impact their quality of life in the future.

The third and final portion of the survey used selected PROMIS® item banks to evaluate general physical health, pain, fatigue, physical function, sleep, depression, anxiety, sexual satisfaction, and social function. Point values for PROMIS® construct responses were calibrated to T-scores generated from a representative U.S. population and reported on a 100-unit scale where the mean is set at 50 units, with 10 units representing one standard deviation (SD). This data transformation follows the guidelines set forth in PROMIS® methodology [18]. Means and 95 % confidence intervals for the OI population were calculated based on individual T-scores for a given concept. The minimum important difference (MID) in PROMIS® responses thought to be clinically relevant has been estimated as 0.3–0.5 SD for fatigue, anxiety and depression; 0.4–0.6 SD for pain; and 0.2 for physical function [19, 20].

Participants accessed the survey through a web-based portal and were recruited with two OIF postal mailings, a notice posted on the OIF web-site, the OIF newsletter *Breakthrough*, spoken and written notification at national and regional clinical and scientific meetings, social networks (Facebook, Twitter, OI forums and chat-rooms), and word of mouth. Children’s National Health System’s Institutional Review Board exempted the survey from review, as it was anonymous. Prior to participation, respondents read the study’s purpose, gained study contact information, learned of the right to withdraw, and then chose either to participate or not participate. The survey instrument was available from 10/01/2011 to 12/31/2011 and designed so that respondents could complete the survey progressively. Those without computer access or facility were offered a paper version, and a data entry specialist masked to subject identity uploaded completed results. Paper surveys were accepted until midnight 01/02/2012. The survey required at least one hour, but more than likely, two hours for completion. Only respondents who answered at least one question in the final PROMIS® item set (Sleep Disturbance) were considered to have completed the entire OI-ANHI survey.

Body Mass Index (BMI) was calculated as kg/m^2^ and classified according to the World Health Organization (WHO) weight classification criteria [21]. Height Z-scores were calculated from the National Health and Nutrition Examination Survey (NHANES III) dataset [22]. Height Z-scores indicate the number of SD a reported height varies from a US adult, gender matched mean. To compare the PROMIS® scores of the generalized population with those of our sample, we segregated PROMIS® T-scores by both OI self-reported severity grouping (mild, moderate, severe) and by quartile of height Z-score. For continuous variables, a t-test was used to calculate the *p*-value; for dichotomous variables, a chi-square test was used. For variables reported on a Likert scale, a generalized linear model was utilized (SAS proc glm). Generalized linear models were also used to compare differences among OI groups. All analyses were performed using SAS version 9.2 (SAS institute, Cary NC).

## Results

**Fig. 1:**
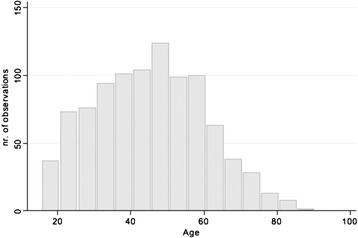
Histogram displaying age distribution for all respondents

**Fig. 2:**
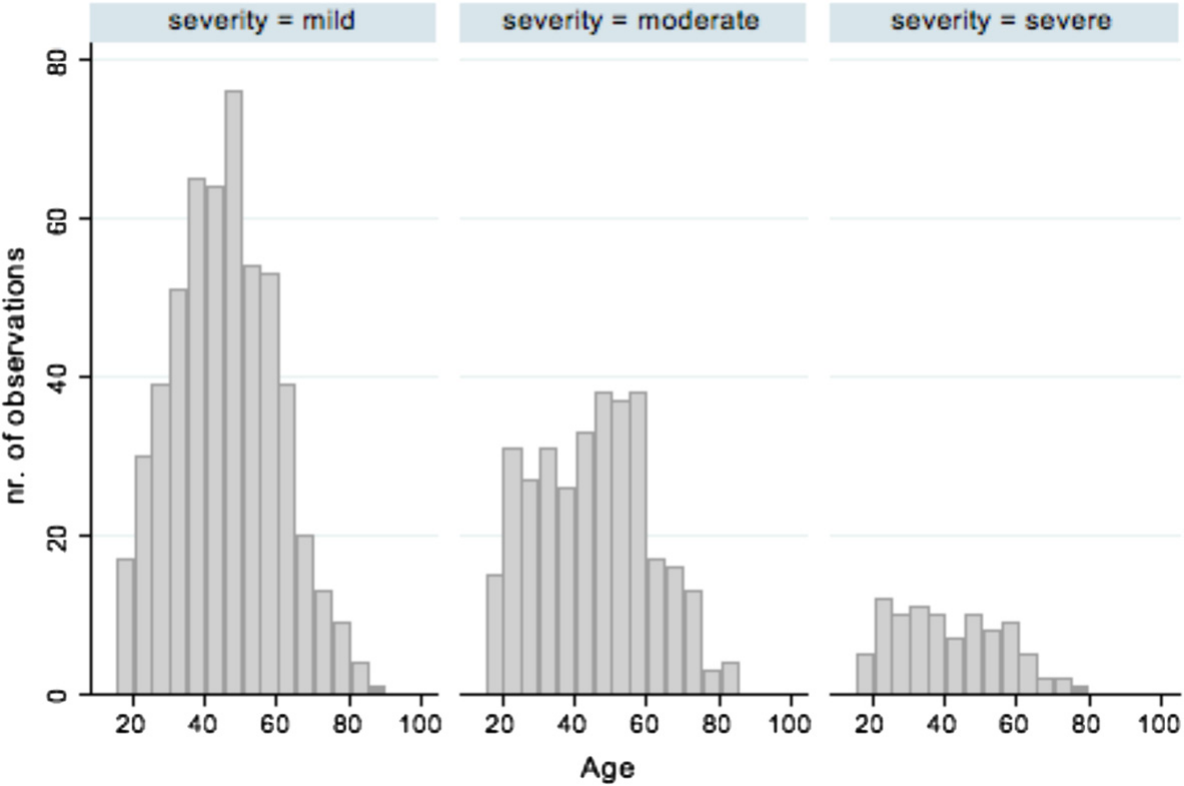
Histogram depicting age in five-year groups by self-reported severity grouping. Three categories of severity are reported: mild, moderate, and severe

Of 1,183 individuals who initiated the survey, 959 (82 %) successfully completed it, constituting the study sample. Demographically, respondents were female (71 %), middle aged (mean 45.1 ± 15.0 years, range 18–89 years), Caucasian (91 %) and lived in North America (89 %). Figure 1 is a graphical depiction of age distribution. Age did not vary on the basis of self-reported severity grouping (Fig. 2). Highest educational achievement exceeded secondary school for 82 %. In regard to anthropometric data, average height was 57.3 ± 9.2 inches, weight 135.9 ± 44.6 pounds, and BMI 29.4 ± 9.3.

**Fig. 3:**
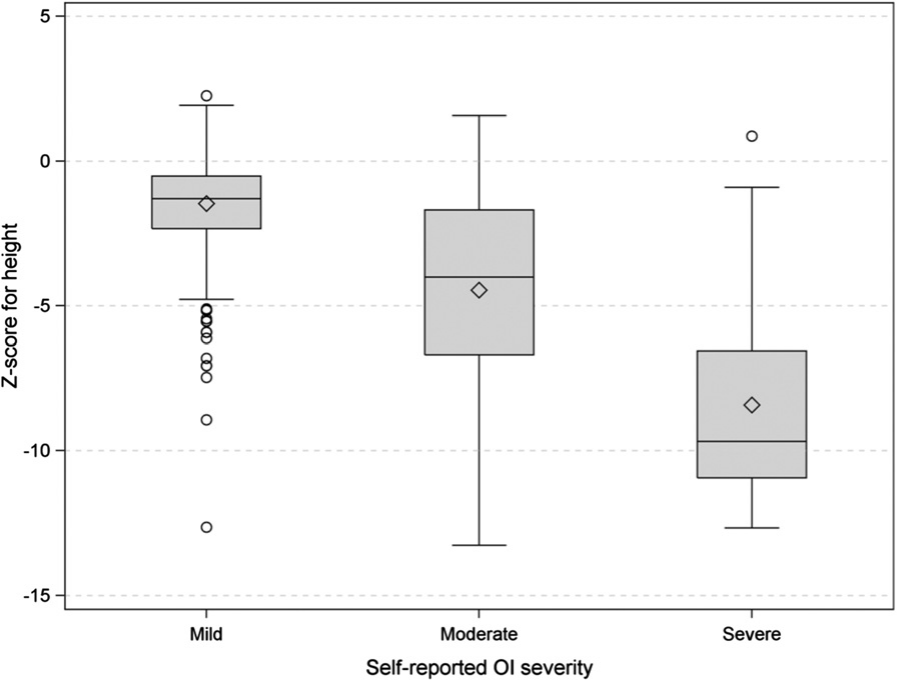
Box plot representing self-reported OI severity and NHANES-III Z-score for height. The diamonds represent the mean and the horizontal lines within the box the median height Z-score. The *upper* and *lower* bounds of the box represents the 75^th^ and 25^th^ percentile for height Z-score respectively. The whiskers represent the minima and maxima height Z-score values and *open circles* represent outliers

In response to the OI-specific medical questions, the majority walked unaided (61 %), but 16 % required a cane, crutches or walker, and nearly one-quarter reported regular wheelchair use. The majority had received their OI diagnosis from a doctor (59 %). Respondents considered their OI to be mild (56 %), moderate (35 %) or severe (9 %) and reported Sillence classifications of Type I (39 %), Type III (13 %), and Type IV (12 %). Fewer than 5 % described other Sillence types and 31 % reported their type to be unknown. Table 1 shows the relationship between self-reported disease severity and self-reported Sillence type. As self-reported disease severity worsened, height Z-score decreased (β = -0.13, *p* < 0.0001, Fig. 3).

**Table 1:** Relationship between self-reported Sillence type and self-reported disease severity in adults with OI

		Self-reported disease severity		
		n (% of OI type)		
OI type	n (%)	Mild	Moderate	Severe
I	369 (39)	308 (83)	59 (16)	2 (<1)
II	26 (3)	12 (46)	8 (31)	6 (23)
III	125 (13)	7 (1)	67 (20)	51(57)
IV	110 (12)	45 (41)	55 (50)	10 (9)
V	14 (1)	2 (14)	11 (79)	1 (7)
VI	2 (<1)	–	1 (50)	1 (50)
Bruck’s syndrome	3 (<1)	–	3 (100)	–
Unknown	295 (31)	154 (52)	123 (42)	18 (6)
Total	944 (100)	528 (56)	327 (35)	89 (9)

Missing *n* = 15

**Fig. 4:**
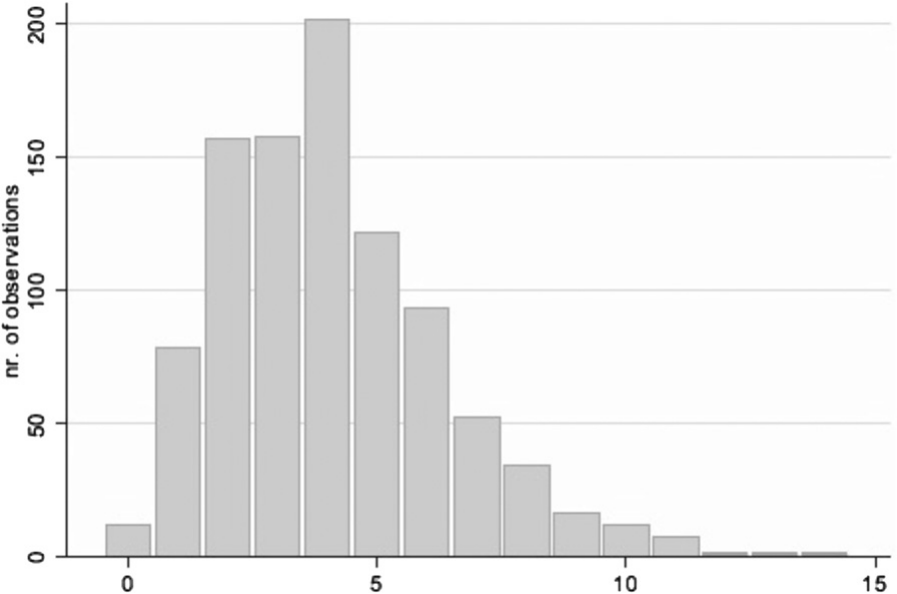
Histogram displaying number of healthcare providers seen for all respondents

A large proportion of participants (65 %) consumed vitamins or dietary supplements, followed by pain (40 %) and blood pressure medications (32 %). Medical providers reported to be actively involved in respondents’ medical care included a “general medical doctor” (92 %), orthopedist (49 %), gynecologist (40 % of women), and endocrinologist (21 %). Cardiologists, dermatologists and gastroenterologists were involved in 10 % of respondents’ care, but few other medical specialists had ever been engaged. Other providers were dentists (68 %) and ophthalmologists (30 %). Both overall and when separated by self-reported severity grouping, respondents saw a median number of 4 different care providers. The overall range was 0 to 14 (Fig. 4). Number of health care providers seen did not vary on the basis of self-reported OI severity grouping. Notably, just over half of participants reported no health interventions (52 %), while 35 % had received rodding surgery. Other than the two aforementioned responses, respondents indicated a scant number of other health interventions. Many of the respondents had received medical testing in the prior year with 86 % reporting a blood pressure reading, 71 % a vision exam, 66 % a blood test for cholesterol, and 34 % a DXA scan. Respondents reported very few cancer diagnoses, with the majority reporting none (90 %) followed by skin cancer (4 %).

**Fig. 5:**
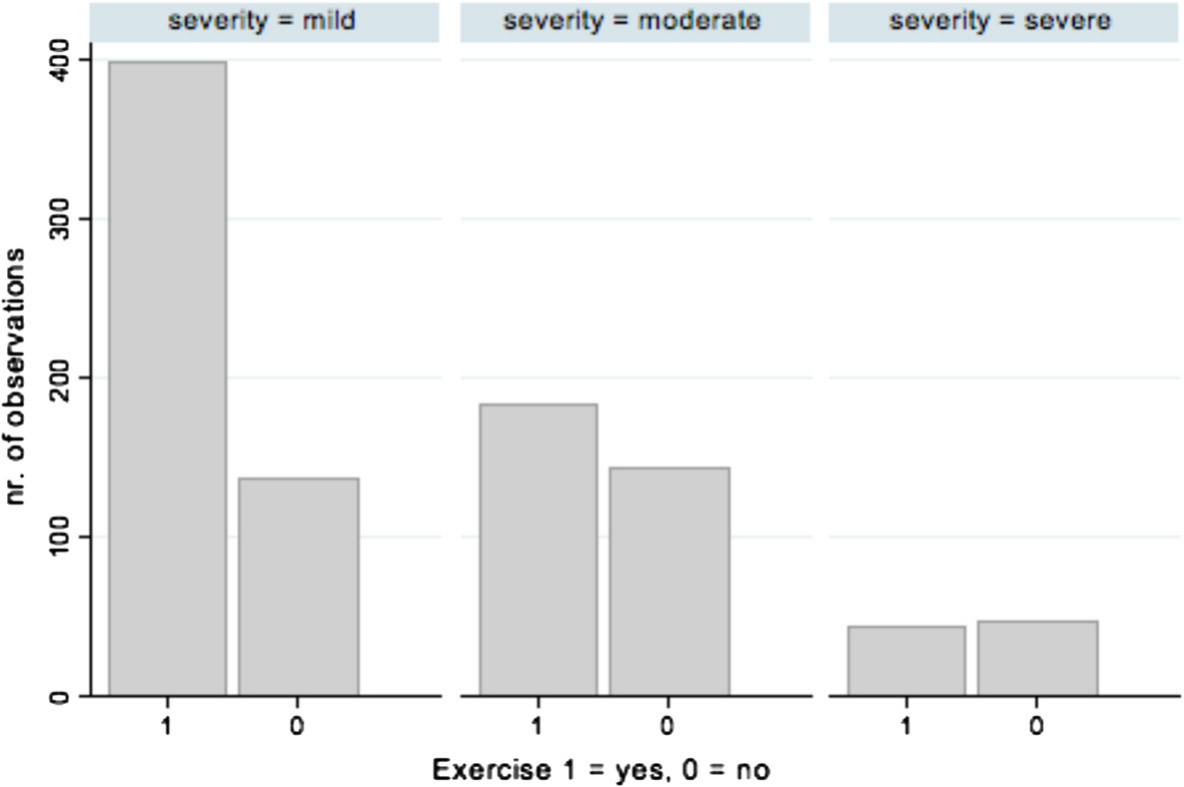
Histogram depicting engagement in or disengagement from exercise by self-reported severity grouping. A response of “1” correlates with “yes” for exercise while “0” represents a response of “no.” Three categories of severity are reported: mild, moderate, and severe

Respondents acknowledged few general health concerns, with “none of these” (52 %) as the most endorsed response followed by changes in sleep patterns (27 %) and mood swings (20 %). Almost half of respondents (49 %) engaged in walking as a regular physical activity although approximately one-third (34 %) reported that they do not exercise regularly. There was a significant relationship between engagement in physical activity and severity grouping (Fig. 5). The most popular exercise venue was the home (51 %), and respondents oftentimes developed their own exercise programs (40 %).

Only 2 % of respondents reported having no medical provider and 4 % considered urgent care or emergency room providers to be their primary source of health care. Ninety-one percent of respondents had health care insurance. Many adults with OI turn to their medical providers for health-related information (53 %); however, more rely on the Internet (63 %) and the OIF (71 %) for that information. Indeed, only 32 % indicated “quite a bit” or “very much” confidence in their primary care provider’s medical management of their OI. Approximately one-third of respondents reported no regular dental care.

Table 2 summarizes prevalence for various self-reported conditions and symptoms, segregated by self-reported disease severity, compared to similar results reported in the NHIS 2012 [23] and other benchmark datasets [24–38]. Musculoskeletal problems (particularly fractures), hearing loss, heartburn, and astigmatism differ most between OI-affected adults and the general population. Reports of dry skin, numbness, sleep apnea, hemorrhoids and shortness of breath appear to be more prevalent in adults with OI. Only fracture, hearing loss and to some extent heartburn and constipation relate to OI severity. In regard to obstetric/gynecological health, an unexpected finding was that one-third of adult women with severe OI reported a prior pregnancy, with only 6 % reporting miscarriage and none reporting stillbirths. Among the women in our sample 55 years of age and younger, those reporting severe OI experienced significantly less (*p* < .05) catamenial bleeding than those with mild or moderate OI.

**Table 2:** Prevalence of self-reported conditions and symptoms by adults with OI shown by self-reported disease severity compared with prevalence reported by both genders in NHIS 2012 or, where specified, in other adult benchmark databases

Condition			Prevalence of reported conditions and symptoms (%)		
	Mild OI	Moderate OI	Severe OI	All	NHIS 2012 [23]
	(*n* = 535)	(*n* = 329)	(*n* = 92)	(*n* = 959)	
Astigmatism	33	37	30	34	3.6 [24]
Blood vessel problem (rupture, aneurysm)	5	6	10	6	1–12 [25]
Bruising	47	41	15	42	18 (12–55)
Cancer	11	9	4	10	5–8 [26]
Cataracts	8	9	11	8	8.6 [25]
Constipation	23	29	34	26	12–19 [27, 28]
Coronary artery disease	6	11	8	4, 7	5–17 (<65 yr)
	20	12	0	10, 10	30 (>65 yr) [25]
Heart attack	6	9	7	7	2.9 (>20 yr) [25]
Cough	11	19	30	16	9–33 [29]
Diabetes mellitus	11	11	8	11	8–10 [23]
Dry skin	35	30	28	32	14–19 (<65 yr)
	49	62	0	52	56 (>65 yr) [30]
Fractures	59	72	66	64	1–3 [31]
Fragile teeth	29	46	57	38	10–19 [32]
Glaucoma	8	7	2	7	2 [24]
Hearing loss	59	57	58	58	2–16 [23]
Heart valve problem	6	9	4	7	2–4 [25]
Heartburn	30	40	30	33	3–7 [33]
Hemorrhoids	17	18	11	17	4 [28]
High cholesterol	29	27	12	26	8–27 [23, 25]
Hypertension	32	42	34	35	21–36 [34]
Kidney/bladder stones	8	14	4	9	8.8 [35]
Near sightedness	50	49	39	49	33–40 [24]
Numbness	18	22	25	20	0.1–8 [36]
Obesity (BMI > 30 kg/m^2^)	29	42	50	35	28–35 [23]
Shortness of breath	20	31	47	26	10–18 (<65 yr)
	28	31	60	31	30 + (>65 yr) [37]
Sleep apnea	9	17	32	14	<6 [38]
Stroke	3	6	8	4	3 [25]
Wheezing/asthma	12	14	27	14	8–13 [37]

NHIS 2012: “Any hearing trouble without use of hearing aid or listening device”

**Fig. 6:**
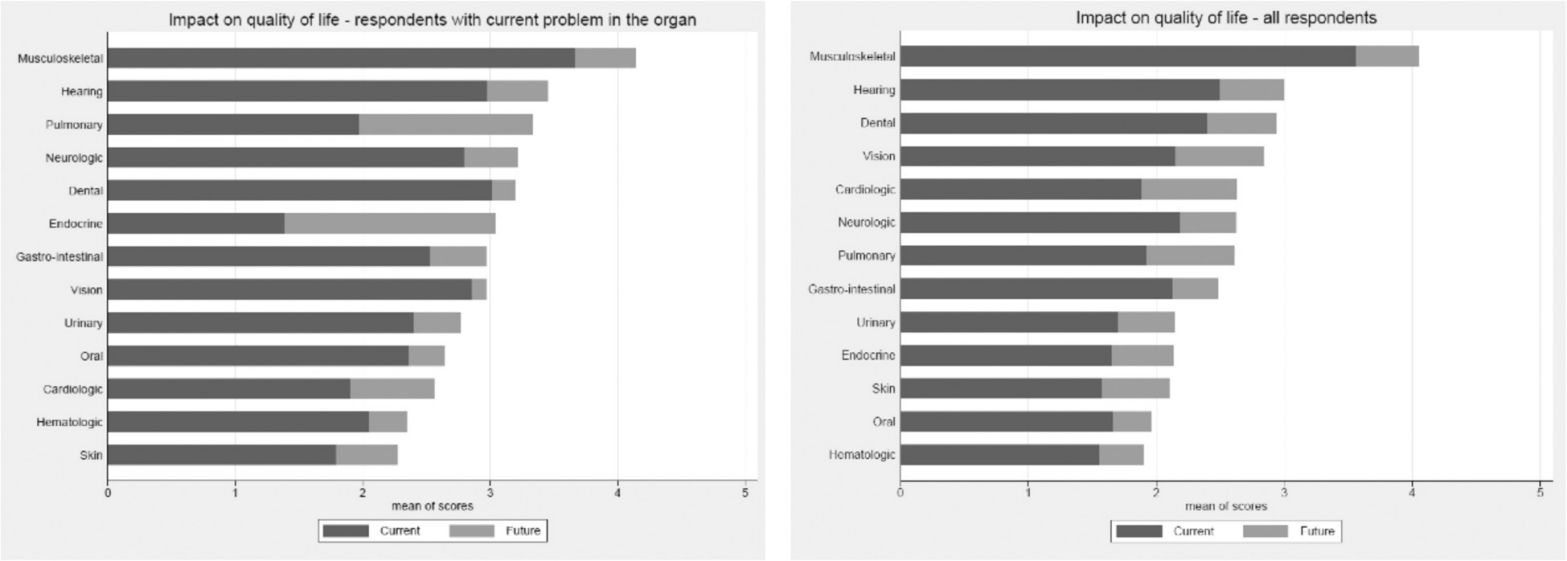
The impact of specific organ system concerns on current and anticipated future QoL for all respondents (*left*) and for those already experiencing problems within that organ system (*right*) described using a 5-point Likert scale ranking of organ-system-based concerns from least (1) to greatest (5) impact on current and anticipated future quality of life

The organ systems most often reported to affect current QoL are urinary tract (97 %), musculoskeletal (95 %), vision (82 %), auditory (75 %), dental (74 %), skin (68 %), gastrointestinal (65 %), and neurological (64 %). Using a Likert scale, Fig. 6 shows the impact of specific organ system concerns on current and anticipated future QoL for all respondents (Fig. 6 left) and for those already experiencing problems within that organ system (Fig. 6 right). Disproportionate concern was reported for future pulmonary, cardiologic and vision issues by all respondents, and for endocrine, pulmonary and cardiovascular issues in those respondents already experiencing issues in these domains.

**Fig. 7:**
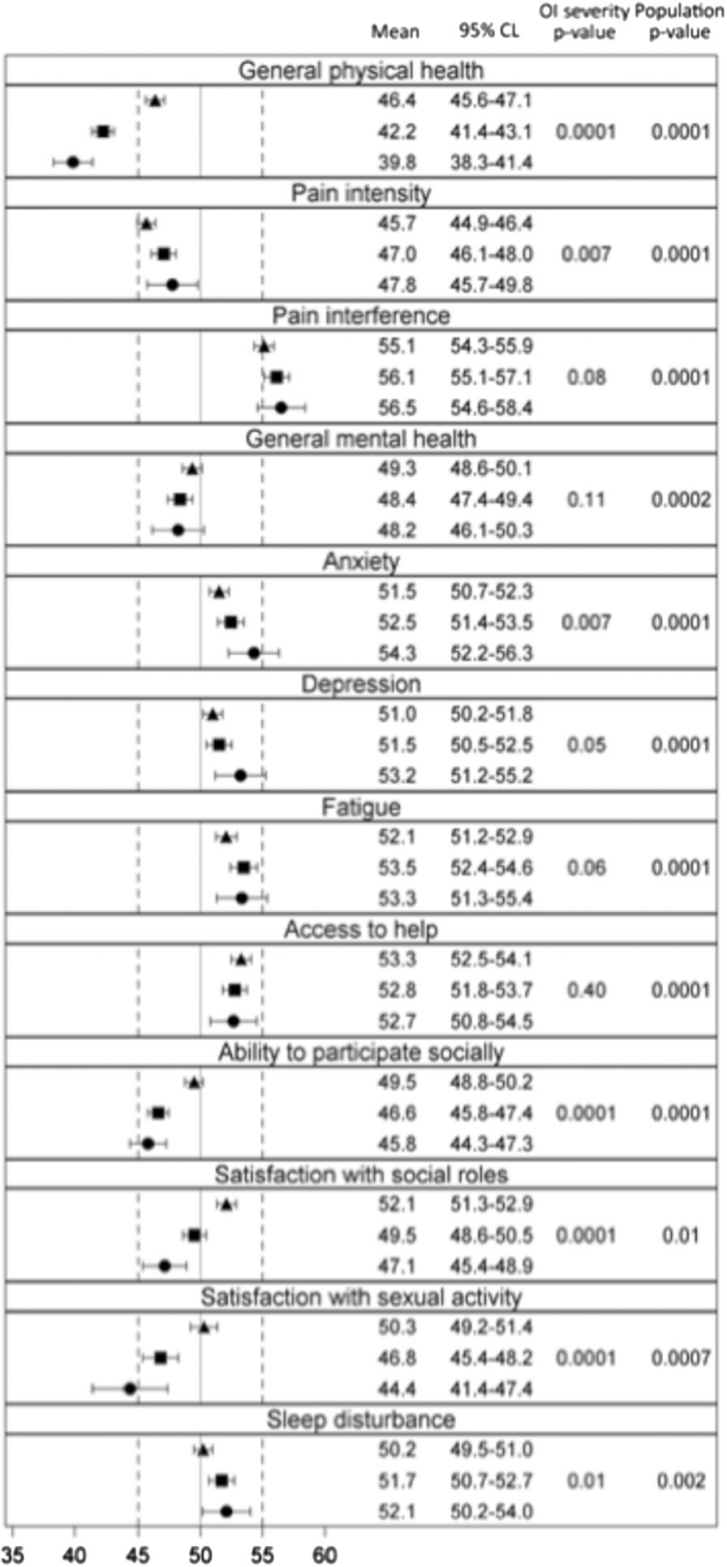
Mean PROMIS® T-scores with 95% confidence limits by self-reported OI severity. Symbols represent self-reported OI severity (▲ = mild, ■ = moderate, ● = severe). The mean T-scores of the PROMIS® referent population are indicated by the solid vertical reference line placed at “50”. Therefore, symbols to the right of the reference line are higher than the PROMIS® population mean (i.e. more of that construct than the PROMIS® population) and symbols to the left of the reference line are lower than the PROMIS® population mean (i.e. less of that construct than the PROMIS® population). *p*-values are for the comparisons of T-scores within disease severity strata amongst the entire OI cohort (*left* column, “OI severity”) and between the entire OI cohort and the general PROMIS® population (*right* column, “Population”)

**Fig. 8:**
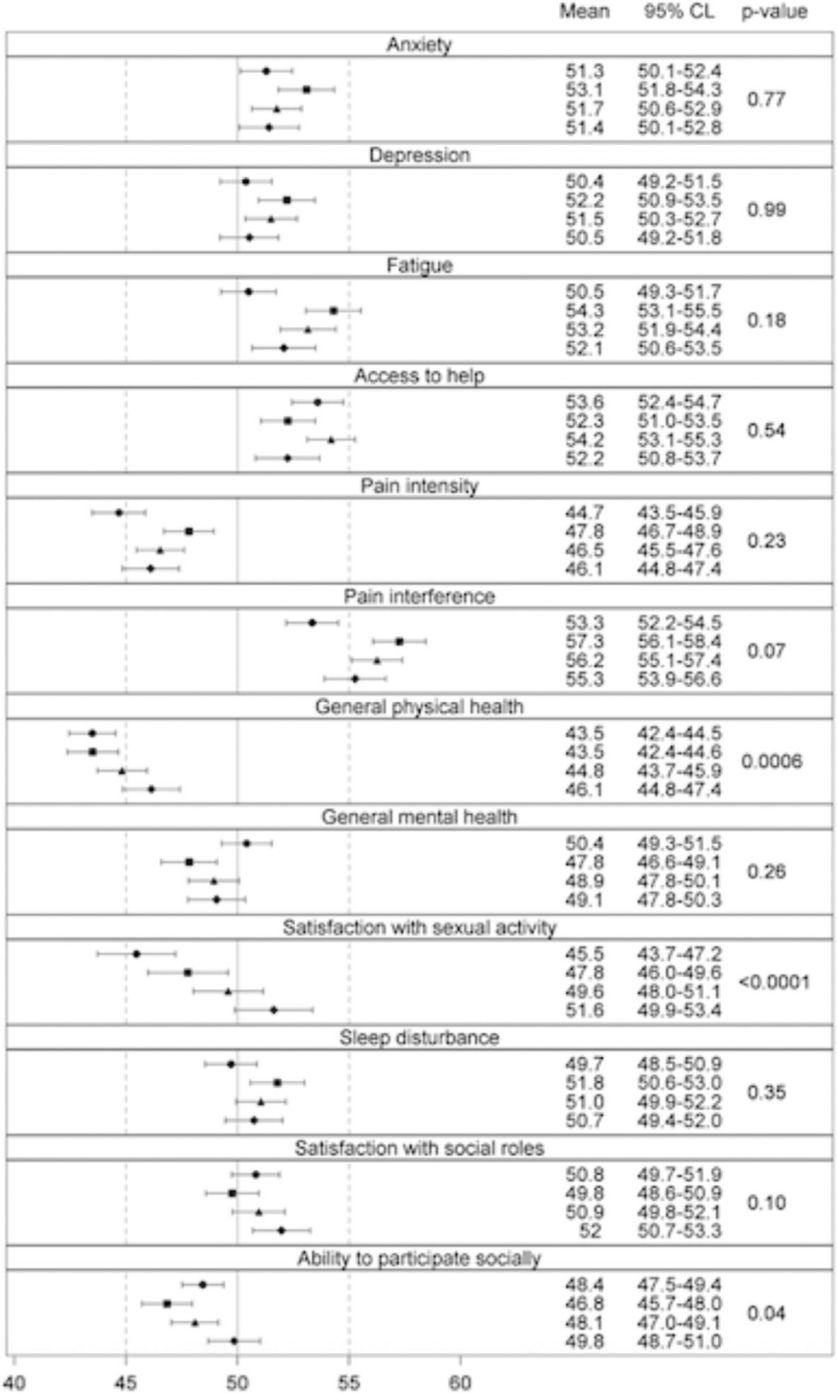
Mean PROMIS® T-scores with 95% confidence limits by quartile of NHANES-III Z-score for height. Symbols represent self-reported OI severity (▲ = mild, ■ = moderate, ● = severe). The mean T-scores of the PROMIS® referent population are indicated by the solid vertical reference line placed at “50”. Therefore, symbols to the right of the reference line are higher than the PROMIS® population mean (i.e. more of that construct than the PROMIS® population) and symbols to the left of the reference line are lower than the PROMIS® population mean (i.e. less of that construct than the PROMIS® population). *p*-values are for the comparisons of T-scores within disease severity strata amongst the entire OI cohort (*left* column, “OI severity”) and between the entire OI cohort and the general PROMIS® population (*right* column, “Population”)

Figure 7 shows the mean PROMIS® T-scores of selected items by self-reported OI severity compared with the PROMIS® population norm. General physical health status was significantly lower than that of the general population and the magnitude of difference (0.4–1.0 SD) exceeded the MID established for several PROMIS® domains [19, 20]. General physical health among the OI cohort was lower in those reporting greatest disease severity but the magnitude of this difference was small. Results also revealed small but statistically significant differences between adults with OI and the normative population for a number of the PROMIS® scales; adults with OI reported greater levels of anxiety (*p* < .0001) and depression (*p* < .0001), as well as lower levels of general mental health (*p* < .0002). However, the differences in means between adults with OI and the normative population for these three scales did not qualify for clinical relevance according to the established MIDs [19, 20]. Segregation of PROMIS® T-scores by quartile of height Z-score (Fig. 8) demonstrates results similar to Fig. 7 and shows that decreased height does not consistently correlate with less desirable PROMIS® scores within our adult OI sample for certain scales, including access to help and satisfaction with social roles.

Remarkably, adults with OI report lower pain intensity than the general PROMIS® population. While adults with OI indicate that pain interferes with their functional state significantly more than it does for the general PROMIS® population, this difference appears to be less than the MID and results did not differ by self-reported disease severity.

Only those adults with severe OI appeared to report notably (-0.56 SD) lower satisfaction with sexual activity. Statistically significant differences in T-scores for responses to “sleep disturbance”, “satisfaction with social roles” “fatigue”, “access to help”, and “ability to participate in social activities” were found between the OI cohort and the general PROMIS® population but these differences appear to be less than a MID.

## Discussion

Delineating and meeting the health needs of adult survivors of childhood-onset conditions is a growing quality-of-care issue. Based on estimates of those living with OI in the US ranging from 25,000 [39] to 50,000 [40], approximately 3 % of the total US OI population completed the OI ANHI survey. This number is especially impressive given the fact that adults with OI are a rare, geographically dispersed population and that we recruited respondents in an incentive-free manner. In doing so, we have demonstrated how persons with rare (and not so rare) conditions can collectively address some of their important health related concerns as a virtual community.

### Demographics and medical/lifestyle information

BMI exceeded the WHO diagnostic threshold for obesity (30 kg/m^2^) in 36 % of respondents and an additional 31 % were overweight. While this prevalence is similar to the general adult US population, it is unclear that this finding conveys the same health risk for an individual whose height is 5 or more SDs below average. Hypertension has been reported to be more prevalent in adults with OI but this was not the case in our population. Unexpectedly, adults with self-reported severe OI reported substantially less catamenial bleeding than those reporting mild or moderate disease. Heightened vigilance for cutaneous trauma or the protective effect of a wheelchair might explain the former but could not account for the latter. While vague, the large number of respondents reporting numbness suggests the need for further inquiry as the incidence of basilar invagination, a potential explanation for numbness, is much higher in persons with OI than the general population.

Neither age nor number of health care providers seen varied by self-reported OI severity grouping. The finding regarding age was surprising as we anticipated that those individuals with more severe forms of OI would be younger because of the higher number of medical and musculoskeletal problems that can accompany more severe disease. While those with the milder types of OI, such as types I and IV, can anticipate a normal lifespan, prior research has found a reduced lifespan for those with type III OI [12, 41].

The findings that age does not vary on the basis of the subjective self-reported grouping and that height is an inconsistent predictor of quality of life suggest the same outcome: there are important aspects of OI severity and functioning not captured by some of the markers relied upon in the past, including age and height. These inconsistencies underscore the need to validate the PROMIS® instruments for use in OI and to develop disease-specific QoL measures in order to better assess functioning in adults with OI. Satisfaction with sexual activity was the only construct that revealed statistically significant differences between groups.

We determined significant differences in exercise engagement based on perceived disease severity. This finding deserves further inquiry. Given that this was a self-reported disease severity rating rather than Sillence classification, do adults who report more severe OI indeed abstain from physical activity because of the severity of their condition or are there individuals with more mild forms of OI reporting greater OI severity because their condition has potentially also worsened from exercise abstinence? Previous research among adults with OI determined a correlation between decreased physical function and ability to participate in the physical actions of climbing stairs and going for walks [8]. Similarly, a study among pediatric patients with OI found that walking ability suffered with greater OI disease severity [42]. Such research suggests that physical activity participation is not only a challenge in OI, but also becomes less likely with augmented OI severity.

However, physical activity, especially physiotherapy involving muscle strengthening, is an important aspect of OI management [8] and physical activity has been shown to improve functioning in pediatric populations with mild to moderate OI [43]. Unfortunately there are no studies to guide persons with severe OI. In light of our finding that adults with self-reported, severe OI show greater disengagement from exercise, future studies should examine this association and its determinants. In turn, longitudinal research should examine the potentially protective effect of exercise by all types of OI disease severity.

### Organ-specific quality of life concerns

Adult OI respondents indicate that musculoskeletal and auditory issues have a greater impact on current and anticipated future QoL than other organ system concerns, and express even greater concern about the future impact of endocrine and pulmonary issues on QoL. Respiratory failure is a leading cause of death in severe OI [12, 41]. The lungs are often greatly affected in OI, with challenges such as pulmonary restrictive disease due to chest wall or pulmonary collagen abnormality, airway obstruction, pulmonary hypertension, and sleep apnea [44]. Care recommendations have been developed by Sandhaus, which include intensive, longitudinal follow-up of pulmonary function in patients with OI [44]. This is particularly important given the high rates of sedentarism and elevated BMI reported [12, 45]. Therefore, the low rate of reported pulmonary consultation could be an opportunity for improving care and health status.

Up to 50 % of adults with OI have dentogenesis imperfecta [6] yet approximately one-third of our respondents reported no regular dental care, and 74 % expressed current QoL concerns regarding their dental health. An important future research direction should include a more in-depth analysis of dental care in OI to establish whether adults with OI have difficulty gaining access to dental care or whether there is another explanation for this shortfall.

Multiple insights into improving research and care were gleaned from the ROS. Results from the QoL portion of the medical ROS revealed that adults with OI have future-related QoL concerns with organ systems identified as troublesome, particularly the musculoskeletal, auditory, and pulmonary subsystems. In addition, respondents expressed future-related, QoL concerns with the endocrine and gastrointestinal organ systems, subsystems that have not been heavily studied in the existing OI literature. For better incorporation of patients’ concerns into care and integration of what doctors know to be important with what matters to patients, exploration of the endocrine and gastrointestinal subsystems represents an important future research direction.

### Patient-centered/patient-reported outcomes: PROMIS®

As according to PROMIS®, adults with OI report lower general physical health status than the general population. General physical health status among respondents reporting the greatest disease severity was lower, but the clinical significance of this finding is uncertain. Numerous statistically significant differences exist among adults with OI of varying severity as well as between adults with OI and the general population, but the clinical significance of these differences is uncertain. Indeed, the PROMIS® instruments suggest that, overall, the OI community does not differ greatly from the general population. Moreover, the results of the OI-ANHI survey support previous findings that adults with OI report a high level of life satisfaction in spite of a significant disease burden which, in our collective experience, the authors would characterize as resilience [7, 8, 46].

We and others have shown significant correlations between disease severity and height Z-score [17], but when PROMIS® T-scores are segregated by quartile of height Z-score, our results suggest that these measures may convey slightly different QoL information in this population. Clinicians encounter adults with ostensibly mild OI who consider their condition to be severe when their only objective manifestation of OI is adult-onset deafness. Perhaps “severity” should also be viewed from the patient’s perspective and not only by height Z-score or Sillence type. Whether nuances in QoL information conveyed by these different measures are important is unknown but, if so, they could be relevant to the development of a disease-specific QoL instrument for OI. The PROMIS® scales are validated and scaled for a healthy, adult population, which represents a challenge in their application to a chronic, rare disease group. Although our results suggest that adults with OI exhibit similarities with the normative population, it is possible that the scales are not capturing some differences within our sample. The validation of PROMIS® instruments for persons with OI will be an important future study focus so that researchers can better understand QoL in OI and move towards the application of PROs both in clinical settings and comparative effectiveness research [47].

### Limitations

Most likely, our recruitment and web-based strategies resulted in a self-selection/enrollment bias. We were likely unable to capture the responses of non-Internet users in this study. Nonetheless, our study represents the first effort to query a large sample of adults with OI regarding their disease burden and QoL. Although our study relied heavily on an Internet rather than a paper format, several studies have quantified the reliability of Internet research studies by comparing them to identical studies conducted with non-internet formats and have found them to be equally as reliable [48, 49]. Internet methods have been shown to be as reliable as paper formats when measuring patient-reported health outcomes for those suffering from chronic health conditions [50].

Our results may be limited by the restricted demographic heterogeneity of our sample. Male gender, lower educational achievement status, and non-Caucasian race were all under-represented. Previous research has shown that women generally rely on the Internet for health and medical information more than men [51]. In addition, as of 2015, those with college education are more likely than those who do not have high school diplomas to use the Internet and the minority groups of African American and Hispanic ancestry are less likely to be Internet users than Caucasians [52]. These noted differences in Internet usage may partially explain why our sample proved to be predominantly female, well educated, and Caucasian. However, previous studies examining OI have also noted a higher proportion of female [7, 8, 38], more highly educated [45], Caucasian [38] respondents, (the majority of whom describe themselves as having type I OI) [38]. Multiple other studies have also reported a middle-aged mean [7, 8, 45]. Thus, our study accessed those members of the OI community most often reported in other research studies. One of our future goals is to reach the populations under-represented in this study, namely men, minorities, and those with a lower level of education.

Another limitation of this study was the challenge of defining disease severity. Even before Sillence developed the current OI classification in 1979, efforts to stratify disease severity had been ongoing and continue to be a challenge because of the expanding clinical and genetic heterogeneity found by researchers. At present, 17 different genetic causes of OI have been identified [53] and it is anticipated that more will be elucidated. Therefore, the effort to define disease severity is a continuous struggle in OI research. Furthermore, misclassification of OI type was possible due to the self-report nature of our research. Indeed, 31 % of respondents were unaware of their Sillence type, and twelve respondents characterized their OI as both Type II and mild, which, until recently, was considered lethal.

In this study, however, we attempted to focus less on Sillence classification, as our goal was greater inclusion of the patient’s perspective and his or her own assessment of functioning and disease burden. Indeed, it has been documented that physicians and patients sometimes disagree on disease severity in chronic diseases. Lack of concordance between physician and patient ratings of disease severity in rheumatoid arthritis is an apt example [54, 55]. Therein, we developed the severity measure for this study in a self-report format. Yet, objective measures of OI severity were not absent from our work. Rather than rely fully on Sillence classification, we included height as an objective marker of disease severity and then examined its relationship with both self-reported disease severity as well as the PROMIS® instruments.

Chief among the study’s strengths is the very large number of respondents and the PCO perspective of this inquiry. The success of this survey in reaching a large number of adults with OI has encouraged our research team to explore new strategies to engage the groups that were underrepresented in the survey, namely men, persons of limited educational achievement, and non-Caucasians.

## Conclusion

The OI-ANHI survey engaged an adult OI community to identify issues of specific health-related QoL concern and to focus future research efforts designed to improve quality of care. Our results revealed that while adults with OI exhibit similarities with the normative population, there are important differences, including a higher prevalence of musculoskeletal and auditory problems and specific QoL concerns regarding the musculoskeletal, auditory, pulmonary, and endocrine systems. Our future research will focus on expanding the range of health care topics pursued in OI research and on incorporating patients’ QoL concerns into effectiveness evaluations of diagnostic and treatment strategies. Finally, we will strive to develop methodologies that allow us to be more inclusive and engage the broader OI community in our future research studies.

## Availability of supporting data

The data supporting the results of this article are housed in the Osteogenesis Imperfecta Foundation.

## Additional file

Additional file 1:
**The OI Adult Natural History Initiative survey (OI-ANHI survey).** (PDF 291 kb)
