# Supplementary material for: Initial report of the osteogenesis imperfecta adult natural history initiative
Source: Orphanet J Rare Dis. 2015 Nov 14;10:146. doi: 10.1186/s13023-015-0362-2 (PMC4650321; doi:10.1186/s13023-015-0362-2)
Supplement: Additional file 1: — The OI Adult Natural History Initiative survey (OI-ANHI survey). (PDF 291 kb) [file 13023_2015_362_MOESM1_ESM.pdf]

The OI Adult Natural History Initiative Survey
(OI-ANHI Survey)

Consent Form

The purpose of this study is to collect information about the general health profile of adults with Osteogenesis Imperfecta. The survey has been designated as IRB-exempt by the IRB of Children's National Medical Center, Washington, DC.

I understand that if I have any questions I can contact Mary Beth Huber at AdultHealth@oif.org or 1-800-981-2663.

I am 18 years of age or older and freely consent to participate. I understand that I am free to withdraw from the study at any time.

I accept ☐

Please complete all applicable registration information below.
All information will be kept confidential.

Ethnicity
- ○ Hispanic or Latino
- ○ Not Hispanic or Latino
- ○ Not Provided

Gender ○ Male ○ Female

Race
- ☐ American Indian or Alaska Native
- ☐ Asian
- ☐ Black or African American
- ☐ Native Hawaiian or Other Pacific Islanders
- ☐ Not Provided
- ☐ Other
- ☐ White

Year of Birth ____________________

Age (in years) ☐

Welcome. Please read each question and then fill in the circle next to the response that best fits your experience.

The next sets of questions focus on issues that individuals with OI often report as impacting their health.

How was your diagnosis of OI confirmed?

- Doctor told me
- DNA (blood test)
- Skin biopsy (collagen)
- Bone biopsy
- I am not sure
- Other

Do you consider your OI to be:

- mild
- moderate
- severe

What type of OI do you have?

_____ I - Type 1

_____ II - Type 2

_____ III - Type 3

_____ IV - Type 4

_____ V - Type 5

_____ VI - Type 6

_____ VII - Type 7

_____ VIII - Type 8

_____ IX - Type 9

_____ Bruck's Syndrome

_____ I Don't Know

What is your best estimate of your weight in pounds? _____________

What is your height in inches? _____________

What is your primary means of mobility?

- ◯ walk unaided
- ◯ walk with a cane
- ◯ walk with crutches or a walker
- ◯ manual chair I push myself
- ◯ manual chair someone else pushes
- ◯ electric wheelchair

What is the highest educational level you completed?

- ◯ Elementary School
- ◯ Middle School
- ◯ High School
- ◯ Some college
- ◯ College (College graduate)
- ◯ Graduate School (Advance Degree)

What country do you live or reside in?
If you live in the USA, please type in "USA". ______________________

Which of the following health care providers do you see for medical care?

- ☐ **General Medical Doctor (internal medicine, general practitioner)**
- ☐ **Endocrinologist**
- ☐ **Cardiologist**
- ☐ **Orthopedist**
- ☐ **Dermatologist**
- ☐ **Gastroenterologist**
- ☐ **Gynecologist**
- ☐ **Urologist**
- ☐ **Pulmonologist**
- ☐ **Neurologist**

- [ ] None

Which of the following health care providers do you see for medical care?

- [ ] Physical Therapist
- [ ] Occupational Therapist
- [ ] Massage
- [ ] Dentist
- [ ] Ophthalmologist
- [ ] Urologist
- [ ] Audiologist
- [ ] Other
- [ ] None

Have you had any of the following health interventions?

- [ ] Laser Eye Surgery
- [ ] Rodding Surgery
- [ ] Cochlear Implants
- [ ] Spinal Fusion
- [ ] Hip Replacement Surgery
- [ ] Knee Replacement Surgery
- [ ] Other Joint Replacement Surgery
- [ ] None of the above

Where do you go to for information about OI?

- [ ] Family
- [ ] Peers or Friends
- [ ] Medical Providers
- [ ] Internet
- [ ] OI Foundation
- [ ] Other

Who do you consider to be your primary care provider?

_____ General Medical Doctor (internal medicine, general practitioner)

_____ Emergency Room

_____ Urgent Care Center

_____ Specialist

_____ Other

How confident are you in your primary care provider's management of your OI health related conditions?

_____ Not at all

_____ A little bit

_____ Somewhat

_____ Quite a bit

_____ Very much

Currently, Do you have any of the following health concerns:

- ☐ unexplained weight loss
- ☐ fever
- ☐ night sweats
- ☐ change in appetite
- ☐ unexplained falls
- ☐ lumps, bumps or masses
- ☐ changes in sleep pattern
- ☐ mood swings
- ☐ adverse reaction to medications or anesthesia
- ☐ none of these

In the past year, have you had any of the following medical tests:

- ☐ Blood Pressure Reading
- ☐ Vision Exam
- ☐ Blood Test for Cholesterol
- ☐ Procto/Colonoscopy Exam

- [ ] DXA Scan
- [ ] None of these

Have you ever been diagnosed with cancer? If so, which type:

- [ ] Leukemia/ lymphoma
- [ ] Breast
- [ ] Uterine
- [ ] Ovarian
- [ ] Prostate
- [ ] Lung
- [ ] Colon
- [ ] Stomach
- [ ] Bladder
- [ ] Kidney
- [ ] Thyroid
- [ ] Skin
- [ ] Brain tumor
- [ ] Bone tumor
- [ ] Other
- [ ] None

Do you do any of these exercises on a regular basis?

- [ ] Walk
- [ ] Run/Jog
- [ ] Lift Weights
- [ ] Swim
- [ ] Yoga
- [ ] Ride a Bike
- [ ] Martial Arts
- [ ] Pilates
- [ ] Other

- [ ] None/Do Not Exercise Regularly

Where do you exercise?

- [ ] Home
- [ ] Gym
- [ ] Pool
- [ ] Physical or Occupational Therapy Center
- [ ] Other
- [ ] Do Not Regularly Exercise

Check any of the following that apply to your exercise program:

- [ ] Includes Muscle/Strength Training
- [ ] Includes Cardiovascular Fitness
- [ ] Includes Stretching
- [ ] Designed by Personal Trainer
- [ ] Designed by Myself
- [ ] Prescribed by Physical/Occupational Therapist
- [ ] Other
- [ ] Not Applicable

Are you currently covered by any of the following types of health insurance or care plans?

- [ ] Employer or Union Provided Health Insurance
- [ ] Directly Purchased Health Insurance
- [ ] Medicare
- [ ] Medicaid, Medical Assistance or any type of government-assistance
- [ ] TRICARE or any type of military health care
- [ ] VA health care
- [ ] Indian Health Service
- [ ] Other health insurance or health plan
- [ ] No health insurance coverage

Currently, do you have health concerns regarding your skin such as:

- [ ] Rashes
- [ ] Bruises
- [ ] Dry Skin
- [ ] Sweating
- [ ] Freckles/Moles/Spots
- [ ] Pressure Sores
- [ ] Skin Fragility (tearing, sores, ulcers)
- [ ] Wound Healing
- [ ] Other
- [ ] None

Currently, how much do issues with your skin impact the quality of your life?

- ( ) Not at all
- ( ) A little bit
- ( ) Somewhat
- ( ) Quite a bit
- ( ) Very much

In the future, how important do you think issues with your skin will be to your health and well-being?

- ( ) Not at all
- ( ) A little bit
- ( ) Somewhat
- ( ) Quite a bit
- ( ) Very much

What other things about the health of your skin are important for us to know?

Currently, do you have health concerns regarding your eyes or vision such as:

- [ ] Near-sightedness
- [ ] Far-sightedness
- [ ] Astigmatism
- [ ] Lazy Eye (amblyopia)
- [ ] Blurry Vision
- [ ] Double Vision
- [ ] Red Eye
- [ ] Glaucoma
- [ ] Cataracts
- [ ] Retinal Bleeding (hemorrage)
- [ ] Retinal Tear or Detachment
- [ ] Other
- [ ] None

Currrently, how much do issues with your vision impact the quality of your life?

- ○ Not at all
- ○ A little bit
- ○ Somewhat
- ○ Quite a bit
- ○ Very much

In the future, how important do you think issues with your vision will be to your health and well-being?

- ○ Not at all
- ○ A little bit
- ○ Somewhat

- ○ Quite a bit
- ○ Very much

What other things about your eyes or vision are important for us to know?

Currently, do you have health concerns regarding your dental health such as:

- ☐ Cavities
- ☐ Gum Disease or Bleeding
- ☐ Toothache
- ☐ Difficulty Chewing
- ☐ Jaw Pain
- ☐ Soft Teeth
- ☐ Breaking or Fragile Teeth
- ☐ Excessive Wearing Away of Teeth
- ☐ Trouble with Dentures
- ☐ Other
- ☐ None

Currently, how much do issues with your dental health impact the quality of your life?

- ○ Not at all
- ○ A little bit
- ○ Somewhat
- ○ Quite a bit
- ○ Very much

In the future, how important do you think issues with dental health will be to your health and well-being?

- ○ Not at all
- ○ A little bit
- ○ Somewhat
- ○ Quite a bit
- ○ Very much

What other things about your dental health are important for us to know?

Currently, do you have health concerns about your breathing or respiratory system such as:

- ☐ **Shortness of Breath**
- ☐ **Cough**
- ☐ **Excessive Sputum**
- ☐ **Coughing Up Blood**
- ☐ **Wheezing (asthma)**
- ☐ **Frequent Colds**
- ☐ **Sleep Apnea**
- ☐ **Pneumonia**
- ☐ **Activity or Exercise Intolerance**
- ☐ **Other**
- ☐ **None**

Currently, how much do issues with your breathing or respiratory system impact the quality of your life?

- ○ **Not at all**
- ○ **A little bit**
- ○ **Somewhat**
- ○ **Quite a bit**
- ○ **Very much**

In the future,

How important do you think issues with your breathing or respiratory system will be to your health and well-being?

- ○ **Not at all**
- ○ **A little bit**
- ○ **Somewhat**

- Quite a bit
- Very much

What other things about the health of your breathing or respiratory system are important for us to know?

Currently, do you have health concerns about your digestion or gastrointestinal system such as:

- [ ] Constipation
- [ ] Diarrhea
- [ ] Reflux/Heartburn
- [ ] Indigestion
- [ ] Abdominal Pain/Stomach Ache
- [ ] Bloating
- [ ] Soiling/Bowel or Bladder Incontinence
- [ ] Hemorrhoids
- [ ] Irritable Bowel Syndrome/Crohn's Disease
- [ ] Diverticulosis/Diverticulitis
- [ ] Rectal Bleeding
- [ ] Other
- [ ] None

Currently, how much do issues with your digestion or gastrointestinal system impact the quality of your life?

- Not at all
- A little bit
- Somewhat
- Quite a bit
- Very much

In the future, how important do you think issues with your digestion or gastrointestinal system will be to your health and well-being?

- Not at all
- A little bit

- ◯ Somewhat
- ◯ Quite a bit
- ◯ Very much

What other things about the health of your digestion or gastrointestinal system are important for us to know?

Currently, do you have health concerns about your passing urine/peeing or urinary system such as:

- ☐ Kidney or Bladder Stones
- ☐ Urinary Tract Infections (UTIs)
- ☐ Difficulty Starting Urine Flow
- ☐ Difficulty Stopping Urine Flow
- ☐ Getting Up at Night to Urinate
- ☐ Blood in Urine
- ☐ Incontinence
- ☐ Urgency
- ☐ Pain During Urination
- ☐ Frequency of Urination
- ☐ Other
- ☐ None

Currently, how much do issues with passing urine or peeing impact the quality of your life?

- ◯ Not at all
- ◯ A little bit
- ◯ Somewhat
- ◯ Quite a bit
- ◯ Very much

In the future, how important do you think issues with passing urine or peeing will be to your health and well-being?

- ◯ Not at all
- ◯ A little bit

- Somewhat
- Quite a bit
- Very much

What other things about passing urine/peeing or the health of your urinary system are important for us to know?

Currently, do you have health concerns about diabetes or other hormonal conditions such as:

- [ ] Diabetes/High Blood Sugar
- [ ] Low Thyroid Function
- [ ] High Thyroid Function
- [ ] Hypoglycemia or Low Blood Sugar
- [ ] Adrenal Function
- [ ] Parathyroid Function
- [ ] Female Hormonal Issues
- [ ] Male Hormonal Issues
- [ ] Other
- [ ] None

Currently, how much do issues with diabetes or hormonal conditions impact the quality of your life?

- Not at all
- A little bit
- Somewhat
- Quite a bit
- Very much

In the future,

How important do you think issues with diabetes or hormonal conditions will be to your health and well-being?

- Not at all
- A little bit
- Somewhat
- Quite a bit
- Very much

What other things about diabetes or other hormonal conditions are important for us to know?

Currently, do you have health concerns regarding the health of your mouth, throat or nose such as:

- [ ] Vocal Cord Problems
- [ ] Voice Problems
- [ ] Sore Throats
- [ ] Trouble Swallowing
- [ ] Sinus Infections
- [ ] Stuffy Nose
- [ ] Nose Bleeds
- [ ] Nasal Masses (polyps)
- [ ] Other
- [ ] None

Currently, how much do issues with your mouth, throat or nose impact the quality of your life?

- ○ Not at all
- ○ A little bit
- ○ Somewhat
- ○ Quite a bit
- ○ Very much

In the future,

How important do you think issues with your mouth, throat or nose will be to your health and well-being?

- ○ Not at all
- ○ A little bit
- ○ Somewhat
- ○ Quite a bit

- Very much

What other things about the health of your nose, mouth or throat are important for us to know?

Currently, do you have health concerns about bleeding or your blood system such as:

- [ ] Bruising
- [ ] Anemia
- [ ] Prolonged Bleeding
- [ ] Other
- [ ] None

Currently, how much do issues with bleeding or blood conditions impact the quality of your life?

- Not at all
- A little bit
- Somewhat
- Quite a bit
- Very much

In the future,
How important do you think issues with bleeding or blood conditions will be to your health and well-being?

- Not at all
- A little bit
- Somewhat
- Quite a bit
- Very much

What other things about bleeding or the health of your blood system are important for us to know?

Currently, do you have health concerns about your heart such as:

- ☐ **Heart Attack**
- ☐ **Chest Pain**
- ☐ **Abnormal Heart Beat (palpitations)**
- ☐ **Heart Murmur**
- ☐ **Coronary Artery Disease**
- ☐ **Heart Valve Problems**
- ☐ **Blood Vessel Problems (ruptures, aneurysms)**
- ☐ **None**

Currently, do you have health concerns about your cardiovascular system such as:

- ☐ **High Blood Pressure**
- ☐ **Low Blood Pressure**
- ☐ **Fainting/Passing Out**
- ☐ **High Cholesterol**
- ☐ **Poor Circulation**
- ☐ **Ankle Edema, Swelling or Dropsy**
- ☐ **Hardening of the Arteries**
- ☐ **Blood Vessel Problems (ruptures, aneurysms)**
- ☐ **Strokes**
- ☐ **Mini-Strokes (TIAs)**
- ☐ **Other**
- ☐ **None**

Currently, how much do issues with your heart or cardiovascular system impact the quality of your life?

- ○ **Not at all**

- ○ A little bit
- ○ Somewhat
- ○ Quite a bit
- ○ Very much

In the Future,
How important do you think issues with your heart or cardiovascular system will be to your health and well-being?

- ○ Not at all
- ○ A little bit
- ○ Somewhat
- ○ Quite a bit
- ○ Very much

What other things about the health of your heart or cardiovascular system are important for us to know?

Currently, do you have health concerns regarding your ears or hearing such as:

- ☐ Hearing Loss
- ☐ Ear Pain
- ☐ Ringing (tinnitus)
- ☐ Stuffy Ears
- ☐ Vertigo
- ☐ Excessive Ear Wax
- ☐ Trouble with Hearing Aid(s)
- ☐ Other
- ☐ None

Currently, how much do issues with your hearing impact the quality of your life?

- ○ Not at all
- ○ A little bit

- ( ) Somewhat
- ( ) Quite a bit
- ( ) Very much

In the future, how important do you think issues with your hearing will be to your health and well-being?

- ( ) Not at all
- ( ) A little bit
- ( ) Somewhat
- ( ) Quite a bit
- ( ) Very much

What other things about your ears or hearing are important for us to know?

Currently, do you have health concerns about your muscle, joints and bones such as:

- [ ] Muscle Pain
- [ ] Stiffness
- [ ] Joint Swelling
- [ ] Arthritis
- [ ] Low Back Pain
- [ ] Upper back Pain
- [ ] Tight Muscles
- [ ] Decreased Range of Motion
- [ ] Joint Noise or Crepitus
- [ ] Rod Migration
- [ ] Scoliosis/Kyphosis
- [ ] Fractures
- [ ] Muscle Spasms after a Fracture

- [ ] Other
- [ ] None

Currently, how much do issues with your muscles, joints or bones impact the quality of your life?

- ( ) Not at all
- ( ) A little bit
- ( ) Somewhat
- ( ) Quite a bit
- ( ) Very much

In the future,
How important do you think issues with your muscles, joints or bones will be to your health and well-being?

- ( ) Not at all
- ( ) A little bit
- ( ) Somewhat
- ( ) Quite a bit
- ( ) Very much

What other things about your muscles, joints or bones are important for us to know?

Currently, do you have health concerns about your nerves or nervous system such as:

- [ ] Headache
- [ ] Seizures
- [ ] Fainting or Spells
- [ ] Dizziness or Dizzy Spells
- [ ] Feelings like Pins and Needles
- [ ] Numbness
- [ ] Weakness
- [ ] Tremor or Shaking

- [ ] **Spasticity (tight or stiff muscles that resist stretching)**
- [ ] **Balance Issues**
- [ ] **Coordination Issues**
- [ ] **Memory Issues**
- [ ] **Attention Issues**
- [ ] **Other**
- [ ] **None**

In the future,
How important do you think issues with your nerves or nervous system will be to your health and well-being?

- ○ **Not at all**
- ○ **A little bit**
- ○ **Somewhat**
- ○ **Quite a bit**
- ○ **Very much**

What other things about your nerves or nervous system are important for us to know?

The next set of questions is only for females.

I am female.

- ◯ Yes, I am female
- ◯ No, I am male

Currently, do you have health concerns about gynecological issues such as:

- ☐ **Irregular Periods**
- ☐ **Heavy Bleeding with Your Periods**
- ☐ **Spotting or Bleeding In Between Your Periods**
- ☐ **Vaginal Discharge**
- ☐ **Frequent Vaginal Infections**
- ☐ **Contraception or Birth Control**
- ☐ **Menopause Symptoms like Hot Flashes**
- ☐ **Other**
- ☐ **None**

Do you have or have you had health concerns about pregnancy or obstetrical issues such as:

- ☐ **I have never been pregnant**
- ☐ **Infertility or Difficulty Getting Pregnant**
- ☐ **Miscarriages**
- ☐ **Stillbirths**
- ☐ **Difficult Labor or Delivery**
- ☐ **Excessive Bleeding During or After Birth**
- ☐ **Pre-Term Labor**
- ☐ **Needed to be on Bedrest**
- ☐ **Other**
- ☐ **None**

Currently, How much do gynecological or obstetrical issues impact the quality of your life?

- ○ Not at all
- ○ A little bit
- ○ Somewhat
- ○ Quite a bit
- ○ Very Much

In the future,
How important do you think gynecological or obstetrical issues will be to your health and well-being?

- ○ Not at all
- ○ A little bit
- ○ Somewhat
- ○ Quite a bit
- ○ Very Much

I have had the following tests or vaccines:

- ☐ **Mammogram in the past 2 years**
- ☐ **Pap Smear in the past 2 years**
- ☐ **Human Papillomavirus vaccine (Gardasil)**
- ☐ **None of the above**

What other things about your gynecological or obstetrical health are important for us to know?

____

The next set of questions is only for males.

I am male.

- ○ Yes, I am male.
- ○ No, I am female.

Currently, Do you have any issues related to your genital organs or sexual functioning such as:

- ☐ **Painful Erection**
- ☐ **Erectile Dysfunction**
- ☐ **Premature Ejaculation**

- ☐ Testicular Pain or Masses
- ☐ Prostate questions or concerns
- ☐ Other
- ☐ None

Currently, How much do issues with your sexual health impact the quality of your life?

- ○ Not at all
- ○ A little bit
- ○ Somewhat
- ○ Quite a bit
- ○ Very much

In the future, How important do you think issues with your sexual functioning will be to your health and well-being?

- ○ Not at all
- ○ A little bit
- ○ Somewhat
- ○ Quite a bit
- ○ Very much

Great progress! The next set of questions focus on aspects of health experienced by adults. These questions are standard health survey questions that have been used on other studies. Remember if you need to take a break, you can stop. Let's get started on this final set of questions!

In general, how would you rate your physical health?

- ○ Excellent
- ○ Very good
- ○ Good
- ○ Fair

- ○ Poor

In general, how would you rate your mental health, including your mood and your ability to think?

- ○ Excellent
- ○ Very good
- ○ Good
- ○ Fair
- ○ Poor

To what extent are you able to carry out your everyday physical activities such as walking, climbing stairs, carrying groceries, or moving a chair?

- ○ Completely
- ○ Mostly
- ○ Moderately
- ○ A little
- ○ Not at all

In the past 7 days How would you rate your pain on average?

- ○ 0
- ○ 1
- ○ 2
- ○ 3
- ○ 4
- ○ 5
- ○ 6
- ○ 7
- ○ 8
- ○ 9
- ○ 10

In the past 7 days how would you rate your pain on average?

- None
- Mild
- Moderate
- Severe
- Very severe

In general, please rate how well you carry out your usual social activities and roles. (This includes activities at home, at work and in your community, and responsibilities as a parent, child, spouse, employee, friend, etc.)

- Excellent
- Very good
- Good
- Fair
- Poor

In the past 7 days How often have you been bothered by emotional problems such as feeling anxious, depressed or irritable?

- Never
- Rarely
- Sometimes
- Often
- Always

Do you have someone to help you if you are confined to bed?

- Never
- Rarely
- Sometimes
- Usually
- Always

Do you have someone to take you to the doctor if you need it?

- Never
- Rarely
- Sometimes
- Usually
- Always

Do you have someone to help with your daily chores if you are sick?

- Never
- Rarely
- Sometimes
- Usually
- Always

Do you have someone to run errands if you need it?

- Never
- Rarely
- Sometimes
- Usually
- Always

I am satisfied with my ability to do things for my family

- Not at all
- A little bit
- Somewhat
- Quite a bit
- Very much

I am satisfied with my ability to do things for fun with others

- Not at all
- A little bit
- Somewhat
- Quite a bit
- Very much

I feel good about my ability to do things for my friends

- Not at all
- A little bit
- Somewhat
- Quite a bit
- Very much

I am satisfied with my ability to perform my daily routines

- Not at all
- A little bit
- Somewhat
- Quite a bit
- Very much

In the past 7 days I felt fearful

- Never
- Rarely
- Sometimes
- Often
- Always

In the past 7 days I found it hard to focus on anything other than my anxiety

- Never
- Rarely
- Sometimes
- Often
- Always

In the past 7 days My worries overwhelmed me

- Never
- Rarely
- Sometimes
- Often
- Always

In the past 7 days I felt uneasy

- Never
- Rarely
- Sometimes
- Often
- Always

In the past 7 days I felt nervous

- Never
- Rarely
- Sometimes
- Often
- Always

In the past 7 days I felt like I needed help for my anxiety

- Never
- Rarely
- Sometimes
- Often
- Always

In the past 7 days How fatigued were you on average?

- Not at all
- A little bit
- Somewhat
- Quite a bit
- Very much

In the past 7 days How run-down did you feel on average?

- Not at all
- A little bit
- Somewhat
- Quite a bit
- Very much

During the past 7 days I have trouble starting things because I am tired

- Not at all
- A little bit
- Somewhat
- Quite a bit
- Very much

In the past 7 days How much were you bothered by your fatigue on average?

- Not at all
- A little bit
- Somewhat
- Quite a bit
- Very much

In the past 7 days To what degree did your fatigue interfere with your physical functioning?

- Not at all
- A little bit
- Somewhat
- Quite a bit
- Very much

I have trouble doing all of my regular leisure activities with others

- Never
- Rarely
- Sometimes
- Usually
- Always

I have trouble doing all of the family activities that I want to do

- Never
- Rarely
- Sometimes
- Usually
- Always

I have trouble doing all of my usual work (include work at home)

- Never
- Rarely
- Sometimes
- Usually
- Always

I have trouble doing all of the activities with friends that I want to do

- Never
- Rarely
- Sometimes
- Usually
- Always

In the past 7 days I felt worthless

- Never
- Rarely
- Sometimes
- Often
- Always

In the past 7 days I felt helpless

- Never
- Rarely
- Sometimes
- Often
- Always

In the past 7 days I felt depressed

- Never
- Rarely
- Sometimes
- Often
- Always

In the past 7 days I felt hopeless

- Never
- Rarely
- Sometimes
- Often
- Always

In the past 7 days I felt like a failure

- Never
- Rarely
- Sometimes
- Often
- Always

In the past 7 days I felt unhappy

- Never
- Rarely
- Sometimes
- Often
- Always

In the past 30 days How satisfied have you been with your sex life?

- Not at all
- A little bit
- Somewhat
- Quite a bit
- Very

In the past 30 days How much pleasure has your sex life given you?

- None
- A little bit
- Somewhat
- Quite a bit
- A lot

In the past 30 days How often have you thought that your sex life is wonderful?

- Never
- Rarely
- Sometimes
- Often
- Always

In the past 30 days How satisfied have you been with your sexual relationship with a partner?

- Have not had a partner in the past 30 days
- Not at all
- A little bit
- Somewhat
- Quite a bit
- Very

In the past 30 days When you have had sexual activity, how much have you enjoyed it?

- Have not had sexual activity in the past 30 days
- Not at all
- A little bit
- Somewhat
- Quite a bit
- Very much

In the past 30 days When you have had sexual activity, how satisfying has it been?

- Have not had sexual activity in the past 30 days
- Not at all
- A little bit
- Somewhat
- Quite a bit
- Very

During the past 7 day I am satisfied with my sex life.

- Not at all
- A little bit
- Somewhat
- Quite a bit
- Very much

In the past 7 days How much did pain interfere with your enjoyment of life?

- Not at all
- A little bit
- Somewhat
- Quite a bit

Very much

In the past 7 days How much did pain interfere with your day to day activities?

- Not at all
- A little bit
- Somewhat
- Quite a bit
- Very much

In the past 7 days How much did pain interfere with your ability to concentrate?

- Not at all
- A little bit
- Somewhat
- Quite a bit
- Very much

In the past 7 days How much did pain interfere with your enjoyment of recreational activities?

- Not at all
- A little bit
- Somewhat
- Quite a bit
- Very much

In the past 7 days

How much did pain interfere with doing your tasks away from home (e.g., getting groceries, running errands)?

- Not at all
- A little bit
- Somewhat
- Quite a bit

- Very much

In the past 7 days How often did pain keep you from socializing with others?

- Never
- Rarely
- Sometimes
- Often
- Always

In the past 7 days How intense was your pain at its worst?

- Had no pain
- Mild
- Moderate
- Severe
- Very severe

In the past 7 days How intense was your average pain?

- Had no pain
- Mild
- Moderate
- Severe
- Very severe

What is your level of pain right now?

- No pain
- Mild
- Moderate
- Severe
- Very severe

In the past 7 days My sleep was refreshing.

- Not at all
- A little bit
- Somewhat
- Quite a bit
- Very much

In the past 7 days I had a problem with my sleep.

- Not at all
- A little bit
- Somewhat
- Quite a bit
- Very much

In the past 7 days I had difficulty falling asleep.

- Not at all
- A little bit
- Somewhat
- Quite a bit
- Very much

In the past 7 days My sleep quality was...

- Very poor
- Poor
- Fair
- Good
- Very good

You have completed the survey! Thank you for your time and participation. Please follow the directions in the cover letter about how to return this to the OI Foundation.
